# Supplementary material for: The Emerging Fusarium graminearum NA3 Population Produces High Levels of Mycotoxins in Wheat and Barley
Source: Toxins (Basel). 2024 Sep 20;16(9):408. doi: 10.3390/toxins16090408 (PMC11435622; doi:10.3390/toxins16090408)
Supplement: Supplementary file 1 [file toxins-16-00408-s001.zip › toxins-3174576-supplementary.pdf]

toxins

MDPI

The Emerging *Fusarium graminearum* NA3 Population Produces High Levels of Mycotoxins in Wheat and Barley

Nicholas A. Rhoades, Susan P. McCormick, Martha M. Vaughan and Guixia Hao

Table S1. Primers and probes used for qPCR amplification.

Oligo name	Organism	Sequence (5' to 3')	Probe
Fg.RED.F	*Fusarium graminearum*	TGACAGCTTTGGTTGTGTTTG	5'-/56-FAM/CGGAAGACT/ZEN/GCTGAGTAACGCCAA
Fg.RED.R		CTTGGCTGGAATGAGTCTGT	
Fg.TEF.F	*Fusarium graminearum*	CAGTCACTAACCACCTGTCAAT	5'-/56-FAM/AACCCAGGC/ZEN/GTACTTGAAGGAACC
Fg.TEF.R		AATGGTGATACCACGCTCAC	
Fg.Tri101.F	*Fusarium graminearum*	GGACTCTGGGATTACGACTTTG	5'-/56-FAM/CGAGACTGT/ZEN/GAGACGGCCAATCTTT
Fg.Tri101.R		ATCAGGCTTCTTGGGCATAAA	
Ta.Ef1.F	*Triticum aestivum*	GATTGACAGGCGATCTGGTAAG	5'-/56-FAM/TCCTCAAGA/ZEN/ATGGTGATGCTGGCA
Ta.Ef1.R		GGCTTGGTGGGAATCATCTT	
Ta.PAL.F	*Triticum aestivum*	GTGTTCTGCGAGGTGATGAA	5'-/56-FAM/AAGCACCAC/ZEN/CCTGGACAGATTGAA
Ta.PAL.R		GTATGAGCTTCCCTCCAAGATG	
Ta.Actin.F	*Triticum aestivum*	CCAAGGCCAACAGAGAGAAA	5'-/56-FAM/TGCCCAGCA/ZEN/ATGTATGTCGCAATC
Ta.Actin.R		GCTGGCATACAAGGACAGAA	
Hv.S.F	*Hordeum vulgare*	CTCCTTGCTTATCCAATTTCTTTTG	5'-/56-FAM/TGGCAAACT/ZEN/GCACCTKTC
Hv.S.R		CTAAGAAGAAACACGGGAAGAAYAA	
horp.F	*Hordeum vulgare*	AGACAAGGCGTGCAGATCG	5'/56-FAM/CCTCAGCCG/ZEN/CAACAGGTG
horp.R		GACCCTGGACGAGCACACAT	
